# A review of published research in the *South African Family Practice* - A clarion call to action

**DOI:** 10.4102/safp.v65i1.5777

**Published:** 2023-07-12

**Authors:** Klaus B. von Pressentin, Ramprakash Kaswa, Shane Murphy, Arun Nair

**Affiliations:** 1 Department of Family, Community and Emergency Care, Faculty of Health Sciences, University of Cape Town, Cape Town, South Africa; 2 Department of Family Medicine and Rural Health, Walter Sisulu University, Mthatha, South Africa; 3 Mthatha General Hospital, Mthatha, South Africa; 4 Outpatient Department, Clinical Services, Abbey House Medical Centre, Navan, Ireland; 5 Department of Family Medicine, University of the Free State, Kimberley, South Africa; 6 Robert Mangaliso Sobukwe Hospital, Northern Cape Department of Health, Kimberley, South Africa

Globally, researchers in primary care and primary health care share the common goals of strengthening the healthcare of their patients and communities through high-quality scholarly practice.^1^ Primary care research is a distinct research discipline and is defined as:

[*R*]esearch on the provision of integrated, accessible health care services by clinicians who are accountable for addressing a large majority of personal health care needs, developing a sustained partnership with patients, and practicing in the context of family and community.^2^

Furthermore, to reflect the need to expand the knowledge foundation in growing our discipline, the term ‘academic primary care’ has been coined to describe the scholarship to draw from and inform excellence in the delivery of primary care.^3^ As a peer-reviewed scientific journal founded in 1980, the *South African Family Practice* (SAFP) journal strives to provide primary care providers and scholars (reflecting both researchers and educators) with a broad range of work in the practice, training and learning of family medicine and primary health care.

Since AOSIS became our publisher in 2020,^4^ we have been able to track and evaluate our journal’s performance metrics. At the time of writing, the journal’s publications had attracted over 4200 citations and 768 600 downloads (2018–2023). The CiteScore has grown from 0.4 in 2012 to 1.1 in 2022 (https://www.scopus.com/sourceid/144682). As editors, we wish to ensure that the original scientific research published in the SAFP makes a relevant contribution to strengthening our knowledge foundation of Southern African primary health care. In line with editorial practices published elsewhere,^5^ we performed a cross-sectional descriptive review of the original research published during 2020–2022. We agreed on key variables, including the coverage of study settings and populations, focus areas and research designs and the typology of primary care research: basic research, clinical research, health services research, health systems research and educational research.^6^ The authors collaborated in capturing the information in a Google form, and data were exported to Microsoft Excel and analysed in Statistical Package for Social Sciences (SPSS) using simple descriptive statistics.

During 2020–2022, 117 original research manuscripts were published. From Table 1^7^, our journal publishes a range of studies across the typology. The clinical research category covers the Southern African quadruple disease burden,^8^ namely, non-communicable diseases including mental health (*n* = 21, 37.5%), communicable diseases including three coronavirus disease 2019 (COVID-19)-related studies (*n* = 10, 17.9%), maternal, newborn and child health-related studies (*n* = 15, 26.8%) and trauma and injury-related disorders (*n* = 5, 8.9%). The study settings appear to be skewed towards research conducted in urban or metropolitan areas with a rural under-representation. Most of the studies focused on adults, with only a handful of studies focusing on children and adolescents. Two-thirds of the published research was quantitative in nature, with descriptive study designs (both cross-sectional and qualitative) representing 91.5% of the published studies. A third of the studies involved cross-institutional collaboration and 40.2% of research teams represented more than one discipline. Half of the first authors were from family medicine and another third were from other medical specialities (including public health). Other primary care-related disciplines were in the minority, such as nursing, pharmacy and rehabilitation sciences. Encouragingly, student-led research represented almost a third of publications, with the majority of these being postgraduate in a 2:1 Master’s to doctoral research ratio.

**TABLE 1:** Findings from a simple descriptive analysis of original research published during 2020–2022 in the SAFP journal (*N* = 117).

Variable	2020 (%)	2021 (%)	2022 (%)	All (%)
**Research publications**				
Original research	34.2	29.1	36.8	100.0
**Primary care research typology**				
Basic research	7.5	32.4	2.3	12.8
Clinical research	47.5	41.2	53.5	47.9
Health services	25.0	17.6	20.9	21.4
Health systems	5.0	0.0	16.3	7.7
Educational research	15.0	8.8	7.0	10.3
**Study setting**				
Urban or metropolitan	70.0	61.8	55.8	62.4
Rural	12.5	17.6	27.6	19.7
Both	17.5	20.6	16.3	17.9
**Study population**				
Adults	77.5	67.6	86.0	77.8
Adolescent	0.0	2.9	0.0	0.9
Children	5.0	0.0	2.3	2.6
Combined	17.5	29.4	11.6	19.8
**Methods**				
Quantitative	75.0	61.8	67.4	68.4
Qualitative	22.5	23.5	23.3	23.1
Mixed methods	2.5	14.7	9.3	8.5
**Study design†**				
Descriptive – survey	70.0	64.7	55.8	63.2
Descriptive – qualitative	22.5	20.6	23.3	22.2
Experimental or quasi-experimental	0.0	2.9	2.3	1.7
Observational analytic	2.5	0.0	4.7	2.6
Research synthesis	2.5	8.8	4.7	5.1
Mixed methods	2.5	2.9	9.3	5.1
**Institutional collaboration**				
Yes	22.5	44.1	32.6	32.5
No	77.5	55.9	67.4	67.5
**Interdisciplinary research teams**				
Yes	47.5	41.2	32.6	40.2
No	52.5	58.8	67.4	59.8
**Discipline of the first author**				
Family medicine	55.0	32.4	58.1	49.6
Other medical specialist disciplines	17.5	32.4	9.3	18.8
Public health	10.0	8.8	18.6	12.8
Nursing	15.0	17.6	2.3	11.1
Education	0.0	5.9	4.7	3.4
Pharmacy	0.0	2.9	2.3	1.7
Rehabilitation sciences	2.5	0.0	0.0	0.9
Social sciences	0.0	0.0	2.3	0.9
Business discipline	0.0	0.0	2.3	0.9
**Country of the first author**				
South Africa	77.5	88.2	93.0	86.3
Eswatini	0.0	2.9	0.0	0.9
Lesotho	0.0	0.0	2.3	0.9
Zimbabwe	2.5	0.0	2.3	1.7
Outside Southern African region but from SSA	12.5	5.9	0.0	6.0
Outside SSA	7.5	2.9	2.3	4.3
**Student-driven research**				
Yes	22.5	20.6	44.2	29.9
**Categories of student-led research (*N* = 35)**				
Undergraduate	0.0	0.0	10.5	5.7
Master’s	33.3	100.0	63.2	62.9
PhD	66.7	0.0	26.3	31.4

*Source:* Grimes DA, Schulz KF. An overview of clinical research: the lay of the land. *The Lancet*. 2002;359(9300):57–61.

SSA, sub-Saharan Africa.

† , The variable ‘Study design’ was informed by Grimes 2022.

This simple descriptive analysis represents a 3-year snapshot of published research in a single South African journal. While the research published here does not represent all primary care and primary health care research performed in our context, this editorial analysis does speak to what is published in our journal and as such we are well positioned to interrogate the strengths and weaknesses of the journal’s research and future goals. The reasons our authors chose the SAFP as a suitable home for communicating their research may be quite diverse. These choices may represent an appreciation for the developmental approaches embraced by the journal’s editors and editorial board, as we are committed to also publishing smaller-scale research from under-researched settings and communities and conducted by early-career researchers, including research conducted as part of postgraduate degrees. However, we are also keen to receive more research submissions from the wider Southern African region, with improved coverage of rural settings, children and adolescents, observational analytical and experimental research and outputs from interdisciplinary teams constituting multiple sites and affiliations.

This editorial serves as a clarion call to the Southern African community of primary care researchers and clinician scholars to review their research focus and help address these persisting research gaps.^9,10^ A multipronged, collective approach is necessary to foster the advancement of primary health care research. The following actions are imperative:

- Increasing access to research funding aligned with primary health care policy commitments.
- Accelerating research capacity-building, through workshops and other interventions.
- Growing scholarly leaders through mentorship as well as enhancing supervisory capacity.
- Establishing interdisciplinary collaborations of research teams representing a range of practitioners, including clinician-scholars embedded in primary health care.

As the SAFP editorial team and board, we are keen to help address these actions in impactful ways. We also plan follow-up analyses of the quality, depth and breadth of research published, by incorporating additional metrics such as the primary health care measurement framework.^11^ Feel free to reach out to us with your ideas and suggestions on how our journal should evolve.
